# Cumulative exposure characteristics of vegetable farmers exposed to Chlorpyrifos in Central Java – Indonesia; a cross-sectional study

**DOI:** 10.1186/s12889-021-11161-5

**Published:** 2021-06-05

**Authors:** Jen Fuk Liem, Muchtaruddin Mansyur, Dewi S. Soemarko, Aria Kekalih, Imam Subekti, Franciscus D. Suyatna, Dwi A. Suryandari, Safarina G. Malik, Bertha Pangaribuan

**Affiliations:** 1 Doctoral Program, Faculty of Medicine Universitas Indonesia, Jakarta, 10430 Indonesia; 2 Department of Occupational Health and Safety, Faculty of Medicine and Health Science Universitas Kristen Krida Wacana, Jakarta, 11510 Indonesia; 3 Community Medicine Department, Faculty of Medicine Universitas Indonesia, Jakarta, 10320 Indonesia; 4 Department of Internal Medicine, Faculty of Medicine Universitas Indonesia, Jakarta, 10430 Indonesia; 5 Department of Pharmacology and Therapeutics, Faculty of Medicine Universitas Indonesia, Jakarta, 10430 Indonesia; 6 Department of Biology, Faculty of Medicine Universitas Indonesia, Jakarta, 10430 Indonesia; 7 Eijkman Institute for Molecular Biology, National Research and Innovation Agency, Jalan Diponegoro No. 69, Kota Jakarta Pusat, Jakarta, Indonesia; 8 Prodia Occupational Health Institute International, Jakarta, 10430 Indonesia

**Keywords:** Pesticide cumulative exposure, Exposure assessment, Exposure reduction, Work practices, Occupational characteristics

## Abstract

**Background:**

Agriculture is a major economic sector in Indonesia. Chemical pesticides are widely being used in agriculture for controlling pest. There is a growing concern that pesticide exposure, particularly chlorpyrifos (CPF) exposure, combined with other occupational characteristics that determine the level of exposure, can lead to further health impacts for farmers. Our objective was to evaluate the cumulative exposure characteristics among farmers exposed to CPF by using a validated algorithm.

**Methods:**

We conducted a cross-sectional study of 152 vegetable farmers aged 18–65 who actively used CPF for at least 1 year in Central Java, Indonesia. Subject characteristics were obtained using a structured interviewer-administered questionnaire, addressed for sociodemographic and work-related characteristics. The cumulative exposure level (CEL) was estimated as a function of the intensity level of pesticide exposure (IL), lifetime years of pesticide use and the number of days spraying per year. CEL was subsequently classified into two groups, high and low exposure groups. The difference in characteristics of the study population was measured using Chi-square, independent-t or Mann-Whitney test. Association between CEL and its characteristics variables were performed by multiple linear regression.

**Results:**

Seventy-one subjects (46.7%) were classified as the high exposure group. The use of multiple pesticide mixtures was common among our study population, with 94% of them using 2 or more pesticides. 73% reported direct contact with concentrated pesticides product, and over 80% reported being splashed or spilt during preparation or spraying activity. However, we found that the proportion of proper personal protective equipment (PPE) use in our subjects was low. Higher volume of mixture applied (*p* <  0.001) and broader acres of land (*p* = 0.001) were associated with higher cumulative exposure level, while using long-sleeved clothes and long pants (*p* <  0.05) during pesticide spraying were associated with lower cumulative exposure after adjusted for age and gender.

**Conclusions:**

These findings indicate an inadequate knowledge of using pesticides properly. Thus, we recommend comprehensive training on pesticide usage and encourage proper PPE to reduce the exposure level.

## Background

Agriculture is a major economic sector in Indonesia. Chemical pesticides are widely being used in agriculture for the control of the pest. Organophosphate (OP) is one of the most widely used pesticides today for that purpose. In 2015, more than half of the pesticides used worldwide were organophosphate (OP) insecticides, with 40% of which were chlorpyrifos (CPF) [1]. The similar situation occurs in Indonesia in the context of the widespread use of pesticides in the agricultural sector [2]. In Indonesia, the number of registered pesticide products has increased from 2605 in 2010 to 3207 in 2016.

Workers in the agricultural sector, especially pesticides applicators, will be exposed to certain amounts of OP and develop certain risks of health problems associated with OP exposure. Generally, exposure to CPF and other pesticides occurs through skin contact, inhalation, or ingestion. Occupational pesticide exposure in the agricultural sector was obtained from several activities, including preparing, mixing, loading, spraying pesticide, and cleaning used equipment. Farmers can also be exposed through re-entering the sprayed area, manipulating crops or harvesting the crops that may still be contaminated with pesticides [3]. Unfortunately, the exposure conditions described above are also accompanied by limited awareness about health problems caused by exposure to pesticides, knowledge of safe work practices, and proper personal protective equipment (PPE) among the farmers [2, 4]. Therefore, there is a growing concern that inappropriate and unsafe use of pesticides may lead to farmers’ health problems [5, 6]. In particular, CPF exposed farmers are vulnerable to several deleterious effects, including neurological symptoms, reproductive hormone alteration, metabolic disruption, and endocrine disruption [7–10].

Several factors such as the type of pesticide, the concentration of the pesticide, the length of exposure, the path of exposure and the proper use of PPE are important factors that determine the severity of the exposure [11–13]. The large-scale experiment in an ideal setting to directly assess the dose-response relationship of pesticides exposure to associated health problems have particular difficulties [12]. Assuming that particular pesticides exposure will lead to specific health problem. In that case, we could expect a linear dose-response relationship between external dose (i.e. occupational and or environmental exposure) and internal dose (i.e. concentration of a chemical or its toxic metabolite in the human body) [13]. This explains that the higher the external dose will result in an increased risk of developing health problems as indicated by the finding of a higher internal dose. However, accurate exposure assessment in epidemiological studies is still difficult to obtain, and real values of exposure to pesticides are not easy to predict [3], especially when resources for assessing direct exposure are limited and studies are conducted in the informal (small-scale) agricultural sector. Therefore, indirect estimation of exposure dose from the worker’s specific task to obtain closer to the actual condition may bridge this gap.

This study’s objective was to evaluate the cumulative exposure characteristics among Javanese vegetable farmers exposed to chlorpyrifos in Indonesia using a validated algorithm. We hope our results will provide supporting data that can be applied to reduce the exposure level for farmers.

## Methods

### Study area and population

We conducted a cross-sectional study of 152 vegetable farmers from 2 villages, Pancot village, Tawangmangu District, and Adipuro village, Kaliangkrik District, which are known as the largest garlic production areas in Central Java, Indonesia from July to October 2020. The agricultural practices and sociodemographic characteristics of farmers in these two villages are very similar to other garlic plantations in Indonesia. There were 92 farmers in Pancot Village and 103 farmers in Adipuro village as the sample frame of the study. From the sample frame, 23 subjects were excluded due to incomplete examination process and 20 other subjects withdrew from the study. The minimum sample size required for this study was 97 subjects, calculated using a single sample formula to estimate proportions with a 95% confidence interval, 10% precision error and 50% estimated CEL proportion. We decided to take the total sample consecutively from the sample frame so that 78 farmers in Pancot village and 74 farmers in Adipuro village who met the criteria and gave written consent to participate in the study were selected as the study subjects. We introduced the study’s objective, goals, and data confidentiality during the recruitment process. Participant enrolment criteria were vegetable farmers aged 18–65 who actively use CPF for at least 1 year.

The study subjects then completed a structured interviewer-administered questionnaire and underwent the anthropometric measurement. Subject characteristics were obtained using a structured interviewer-administered questionnaire addressed for sociodemographic and occupational characteristics. Sociodemographic characteristics consist of several questions such as age, gender, smoking habit and educational background. The interview for agricultural work-related (occupational) characteristics consisted of several specific questions on those related to pesticides exposure, work practice, and the use of personal protective equipment (PPE). We randomly asked several important questions to determine the answer’s consistency to limit the possibility of misclassification of exposure. We also provided short education to raise awareness about pesticide use and safety precautions to the participants at the end of the sessions.

All methods were performed in accordance with the relevant guidelines and regulations. The study protocol was approved by the Ethical Committee of the Faculty of Medicine Universitas Indonesia on March 23, 2020 (No. KET-339/UN2.F1/ETIK/PPM.00.02/2020).

### Cumulative exposure level

The intensity level of pesticide exposure was calculated using the validated method from Dosemeci [14]. The overall exposure intensity level is then combined with information on lifetime years of pesticide use and the number of days spraying per year to produce the cumulative exposure level as shown in the following algorithm:

**IL = (Mix + Appl + Repair + Wash) x PPE x Repl x Hyg x Spill**

IL = Intensity Level of pesticide exposure

Mix = Pesticide mixing activity

Appl = Application methods

Repair = Repairing equipment

Wash = Washing equipment after spraying

PPE = Personal Protective Equipment utilisation

Repl = Replacing old gloves

Hyg = Personal hygiene practices

Spill = Spill treatment (changing clothes after a spill)

There are several similar conditions among study participants in terms of exposure during crop insecticides application. The activities of preparing, mixing, loading, and spraying pesticide using a knapsack sprayer are carried out personally by each study participant in the open area. The status of mixing activity was given a score of 9 for self-preparation and mixing the pesticides; and a score 9 for applying pesticides using a knapsack sprayer. Washing pesticide equipment after spraying was defined as “do not wash” (score = 0) and “rinse tank” (score = 1).

The status of personally repaired spraying equipment was defined as “no repair” (score = 0) and “repair” (score = 2). For the status of replacing old gloves, all participants were given a score of 1.2 for not wearing gloves or using damaged gloves.

The use of PPE use was categorized into the following levels:

- Score 1: Not using PPE
- Score 0.8: PPE-1 (dust mask / goggles / apron)
- Score 0.7: PPE 2 (cartridge respirator / boots)
- Score 0.6: PPE 3 (chemical gloves)
- Score 0.5: Combination of PPE 1 & 2
- Score 0.4: Combination of PPE 1 & 3
- Score 0.3: Combination of PPE 2 & 3
- Score 0.1: Combination of PPE 1, 2 & 3 (proper PPE use)

Personal hygiene habits were scored as follows:

- Score 0.2: Change clothing + handwash/ shower immediately after exposure
- Score 0.4: Change clothing + handwash/ shower at lunch (breaktime)
- Score 0.6: Change clothing + handwash/ shower at lunch (breaktime) or at the end of the day
- Score 0.8: Change clothing + handwash/ shower at the end of the day

Spill treatment (changing clothes after spill) was categorized into 4 levels: changing clothes right away after spill, at lunch, at the end of the day, and at the end of the next day; the scores were 1.0, 1.1, 1.2, and 1.4, respectively.

**CEL = IL x Duration x Frequency**

CEL = Cumulative Exposure Level

IL = Intensity Level of pesticide exposure

Duration = Lifetime years of pesticide use

Frequency = Number of days spraying per year

Because the CEL was not normally distributed, it was classified into two groups, high and low exposure groups, with the median as the cut-off point.

### Agricultural work-related characteristics

There are several agricultural work-related (occupational) characteristics that were not used in the CEL calculation including:

- Arable land area = total arable land area in acres
- Number of arable lands
- Daily work duration (hours) = average duration of all agricultural activities on the farm in hours/day
- Duration of spraying pesticide = average duration of spraying activity in hours/day
- Volume of the mixture applied = average volume of the mixture applied in litre/day

We also categorized the type of knapsack sprayer used as manually pressurised sprayer or motorised sprayer; spraying time as spraying in the morning time or in other time; and the mixture of pesticide as using more than 3 pesticide in mixture or using 1–3 pesticides in mixture.

### Statistical analysis

All analysis was performed using SPSS 20 for Windows.

The study population characteristics were summarised with frequency distribution and percentages for categorical variables, while continuous variables were described using mean ± SD or median (minimum-maximum). Chi-square test, independent t-test or Mann-Whitney test were used to measure the difference in the characteristics of the study population according to the cumulative exposure level group. All *p* values are two-sided, with significance was considered at *p* <  0.05 for these tests.

Association between CEL and its characteristic variables were performed by multilinear regression analysis. Variables associated with CEL at a significance level of *p* ≤ 0.20 in the simple regression analysis were included in the multivariate model. The variables were retained in the final model when they were associated with CEL at a significance level of 0.05 according to the stepwise procedure.

## Results

Our study population was 152 farmers with the mean age of 49.91 ± 9.42 years, consisting of 90.1% male, 92.8% as members of farmers’ society, and 86.8% in low educational level. The median (minimum-maximum) IL score, lifetime years of pesticide use, number of days spraying per year and CEL score (in thousands) were 11.5 (1–23.0), 25 (1–45), 104 (37–364), and 25.9 (0.4–136.6), respectively.

As shown in Table 1, seventy-one farmers (47%) out of 152 were classified as having a high CEL. The proportion of smokers was 48% and significantly higher in the high CEL group. Ten out of 132 subjects in low educational level had never attended formal education.

**Table 1:** Sociodemographic characteristics, lifestyle factors and physical condition of CPF exposed farmers grouped according to the cumulative exposure level

Variable	Cumulative Exposure Level		***p***-value
	High (***n*** = 71)	Low (***n*** = 81)	
Age (years) (mean ± SD)	51.49 ± 8.6	48.52 ± 9.92	0.052^tt^
Member of farmer’s society (n %)	66 (93)	75 (92,6)	0.931^cs^
Male (n %)	65 (91,5)	72 (88,9)	0.583^cs^
Low educational level (n %)	65 (91.5)	67 (82.7)	0.108^cs^
Smoker (n %)	41 (57,7)	32 (39,5)	0.025^cs^
Obese (n %)	16 (22,5)	14 (17,3)	0.417^cs^

*tt* independent t-test, *cs* chi-square

Significantly few farmers reported using pesticides according to the user instructions (2.0%), and all of them were in the low CEL group. As shown in Table 2, the high exposed group was characterised with a broader arable land area, longer daily working time, longer duration of spraying pesticides, and higher volume of mixture applied than the low exposure group. On the other side, the proportion of farmers who used more than three pesticides in the mixture was higher in the low exposure group. The number of days spraying per year was considered high, with > 100 days per year on average.

**Table 2:** Agricultural work-related characteristics of CPF exposed farmers grouped according to the cumulative exposure level

Variable	Cumulative Exposure Level		***p***-value
	High (***n*** = 71)	Low (***n*** = 81)	
Arable land area (acres)^a^	0.25 (0.03–0.70)	0.15 (0.01–0.50)	0.001
Number of arable lands^a^	4 (1–13)	3 (1–9)	0.026
Daily work duration (hours)^a^	7 (3–10)	6 (3–10)	0.003
Spraying in the morning time (n %)	48 (67.6)	44 (54.3)	0.095^cs^
Duration of spraying pesticide (hours/day)^a^	0.57 (0.14–2.00)	0.30 (0.04–2.25)	< 0.001
Volume of the mixture applied (litre/day)^a^	27.2 (7.0–81.6)	14.6 (2.3–85.0)	< 0.001
Used more than 3 pesticides in mixture (n %)	15 (21.1)	30 (37.0)	0.032^cs^
Using a manually pressurised sprayer (n %)	13 (18.3)	23 (28.4)	0.145^cs^

^a^Median (minimum-maximum) with *p*-value by Mann-Whitney test; *cs* Chi-square

There are numbers of similar conditions among study participants in terms of exposure during crop insecticides application. Preparing, mixing, loading, and spraying pesticide using a knapsack sprayer are carried out personally by each study participants in the open area. None of the subjects wore a respirator, coverall, or disposable outer work clothes. The proportion of aprons, goggles, and chemical gloves users in our study population was also tiny (Table 3). However, most of them frequently use long-sleeved clothes or long pants during farm work. Four subjects who used chemical gloves in pesticide exposed activity reported that gloves were only replaced when they were damaged and even then, they often continue using the damaged gloves.

**Table 3:** Distribution of PPE usage, work clothes and work practices of the study population

Variable	Frequency - n (%)	
	Rare / never	Frequent
**Personal Protective Equipment**		
Apron	150 (98.7)	2 (1.3)
Face mask	79 (52.0)	73 (48.0)
Goggles	151 (99.3)	1 (0.7)
Chemical gloves	148 (97.4)	4 (2.6)
Boots*	64 (42.1)	88 (57.9)
**Work clothes**		
Long-sleeved clothes	15 (9.9)	137 (90.1)
Long pants	14 (9.2)	138 (90.8)
**Work practices**		
Wiping sweat with work clothes	123 (80.9)	29 (19.1)
Re-enter the field after spraying	119 (78.3)	33 (21.7)
Spraying against the wind	151 (99.3)	1 (0.7)
Splashed or spilled during spraying	19 (12.5)	133 (87.5)
Splashed or spilled while loading the pesticide	28 (18.4)	124 (81.6)
Eat in the middle of the work-time	147 (96.7)	5 (3.3)
Direct contact with pesticides	41 (27.0)	111 (73.0)
Proper shower after spraying	12 (7.9)	140 (92.1)
Changing clothes after spraying	7 (4.6)	145 (95.4)

*p = 0.001 by chi-square, indicates lower proportion of frequent users in high cumulative exposure group

All of the CPF used were in liquid form, with the majority using a concentration of 200 EC (98.7%). Ethylene-bis-dithiocarbamate (EBDC) mancozeb and abamectin was the fungicide and insecticide most frequently used as an addition to CPF in our subjects, as shown in Table 4. Nearly 2 out of 3 additional pesticides used were in liquid form.

**Table 4:** The proportion of the type of pesticide used besides chlorpyrifos among the study population

Active ingredient	Chemical class	Utilisation	Frequency (n %)
Mancozeb	Carbamate	Fungicide	87 (57.2)
Abamectin	Avermectin	Insecticide	57 (37.5)*
Difenoconazole	Triazoles	Fungicide	49 (32.2)
Emamectin	Avermectin	Insecticide	17 (11.2)
Lambdacyhalothrin	Pyretroid	Insecticide	14 (9.2)
Chlorfenapyr	Pyrrole	Insecticide	12 (7.9)
Beta-cyfluthrin	Pyrethroid	Insecticide	10 (6.6)
Lufenuron^a^	Benzamida	Insecticide	9 (5.9)
Methomyl	Carbamate	Insecticide	9 (5.9)
Fipronil	Phenylpyrazole	Insecticide	7 (4.6)
Dimethoate	Organophosphate	Insecticide	6 (3.9)
Imidacloprid	Neonicotinoid	Insecticide	6 (3.9)
Propineb	Carbamate	Fungicide	6 (3.9)
Deltamethrin	Pyrethroid	Insecticide	4 (2.6)
Profenofos	Organophosphate	Insecticide	4 (2.6)
Cypermethrin	Pyretroid	Insecticide	4 (2.6)
Chlorantraniliprole	Diamide	Insecticide	3 (2.0)
Acephate	Organophosphate	Insecticide	2 (1.3)
Dimehypo	Thiosultap	Insecticide	1 (0.7)
Chlorothalonil	Chloronitriles	Fungicide	1 (0.7)
Mefenoxam	Phenylamides	Fungicide	1 (0.7)
Pyraclostrobin	Carbamate	Fungicide	1 (0.7)
Phoxim	Organophosphate	Insecticide	1 (0.7)
Methoxyfenozide^b^	Benzohydrazide	Insecticide	1 (0.7)
Spinetoram^b^	Spinosyn	Insecticide	1 (0.7)

*p = 0.01 by chi-square, indicates lower proportion of users in high cumulative exposure group

^a^ Product contains a mixture of Lufenuron + Emamectin

^b^ Product contains a mixture of Methoxyfenozide + Spinetoram

**Fig. 1:**
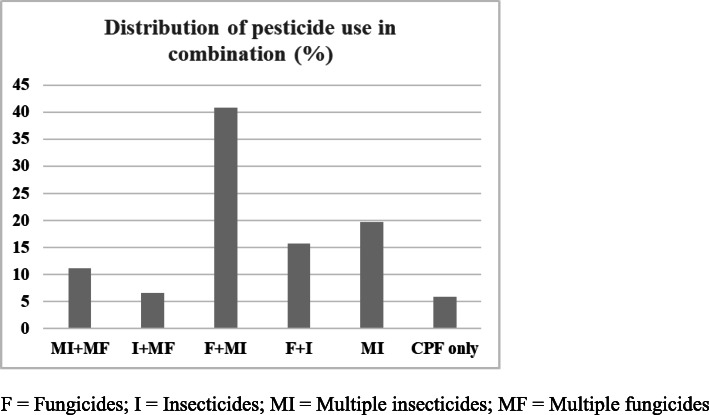
Distribution of pesticide use in combination among study population

As shown in Fig. 1, the use of multiple pesticides is common in our study population. Only 5.9% of the farmers used a single pesticide (CPF) while the other 27, 38.2, and 28.9% used 2, 3, and more than 3 pesticide mixtures.

Using long-sleeved clothes and long pants while spraying, pesticides were associated with lower cumulative exposure while the higher volume of mixture applied and broader acres of land were associated with higher cumulative exposure level after adjusted for age and gender (Table 5).

**Table 5:** Multiple linear regression analysis of cumulative exposure

Variable	B	SE (B)	Beta	95% CI (Lower; Upper)	***p***
Age (years)	0.791	0.165	0.319	0.47; 1.18	< 0.001
Arable land area (acres)	52.633	15,437	0.289	22.12; 83.14	0.001
Volume of the mixture applied (L/day)	0.329	0.107	0.259	0.19; 0.54	0.002
Long pants (work clothes)	−5.691	2.478	−0,160	−10.59; − 0.79	0.023
Long-sleeved clothes (work clothes)	−4.834	2111	−0,160	−9.01; − 0.66	0.023

*B* Parameter estimate, *SE (B)* Standard error for B

Coding for the use of work clothes are as follows: 0 = never, 1 = rarely, 2 = often, 3 = always

R^2^ = 0.361; Adjusted R^2^ = 0.339

## Discussion

In general, farmers in our study have lived most of their lives in this occupation. For them, farming methods and work practices have been taught and implemented over many years. The high number of spraying days per year and the use of multiple pesticide mixtures, while not using proper PPE during agricultural activities is a common practice among them.

Our study showed that the high exposure group’s intensity level was significantly higher compared to the low exposure group due to the significantly higher scores for PPE utilisation, personal hygiene practices, and spill treatment. Since proper PPE utilisation was significant in the exposure reduction strategy, choosing not to use proper PPE will result in a higher internal dose. Several studies have covered the issue that proper use of PPE was significantly associated with lower dimethyl metabolites [15], lower DAP concentrations [16] and the use of full-body coveralls during pesticides handling and spraying was significantly associated with lower OP metabolites level [17].

Dermal exposure and inhalation are the main routes of exposure for agricultural pesticides exposure [11]. All of the CPF used in our subjects were in emulsifiable concentrates that are readily absorbed through skin contact. Thus, direct contact should be avoided, and proper dermal protectors such as chemical gloves, coverall, or apron will reduce the exposure dose [18]. Among our subjects, 73% had reported direct contact with concentrated pesticides product, and over 80% had reported being splashed or spilt during preparation or spraying activity. Contradictory, we found that the most frequently used PPE in our study population were face mask (cloth masks or surgical masks) and boots which did not provide sufficient protection against CPF exposure. However, we also found that appropriate clothing (i.e. long-sleeved clothes and long pants) while spraying pesticides were associated with lower cumulative exposure. These findings are relevant to reduced exposure because long-sleeved clothes and long pants provide a partial barrier against direct contact due to splashes or spills [11, 18]. The proportion of proper PPE use in our study population was 2% while the proportion of ‘no PPE used’ was 15%. The similar condition of low frequency of PPE use has been reported by several studies with agricultural workers in different countries [4, 19–24].

The Hierarchy of Controls defined by NIOSH begins with the most effective measures which are eliminating the hazard, followed by substitution, engineering controls, administrative controls, and the least effective controls, the PPE [25]. The elimination, substitution, and engineering controls will be very difficult to implement in the informal agricultural settings, leaving only 2 options. Many people rely on PPE as the last resort. However, it is generally accepted that advising the use of PPE alone does not always result in adequate protection [26]. For that, the administrative control to change the way they work has to be put in place together with the use of PPE. Regarding the hygiene practices and spill treatment, we found that nearly all of our subjects reported having proper showering and changing clothes after spraying, just in agreement with the results from other researchers [4, 27]. All farmers also claim to wash their hands after being exposed to pesticides and before eating. We suggested this represents a more general attitude rather than acceptable practices in exposure reduction as reported in the previous study in Iran and Indonesia that there is no consistency between perception and work practices [4, 21]. Nevertheless, changing clothes was found to be significantly associated with lower exposure levels, so this practice is as crucial as PPE utilisation, especially to control dermal exposure [17].

Our study also found that very few farmers reported the use of pesticides according to the user instructions. A previous study reported that the level of education promoted safety behaviours among farmers [21, 28, 29]. Farmers with higher education, in general, are having a good sense of safety behaviours during pesticides handling. Higher education will also help farmers to obtain relevant knowledge of work practices and choose the proper PPE [30]. Regarding the use of pesticides, 94% of our subjects reported using two or more pesticides. The high frequency of farmers using multiple pesticides was also found in other countries [19, 23]. Ethylene-bis-dithio-carbamate (EBDC) mancozeb and abamectin were the fungicide and insecticide most frequently used in our subjects, similar to the previous study [31].

Our findings provide a clearer picture of the Javanese farmers’ characteristics in the informal agricultural sector in Indonesia and may also represent conditions in other countries.

There are some limitations to our study that should be taken into consideration while interpreting our results. All of the information regarding agricultural activities were self-reported by the farmers that may result in exposure misclassification. However, the possibility of misclassification has been limited by randomly asking several important questions to determine the answers’ consistency. There are several parameters related to exposure doses that we could not get in the interview. We did not have information regarding the exact quantity of CPF or other pesticides used by the farmers. We also did not have the exact information about the length of time for proper showering or thorough hand washing after direct exposure or after pesticides handling.

## Conclusions

In conclusion, despite the above limitations, the results showed that our study population was characterised by the low frequency of PPE usage, especially the use of dermal protectors and poor work practices (i.e hygiene practices and spill treatment). As an addition to CEL, the occupational characteristics such as a higher volume of mixtures applied, broader arable areas, and extra coverage work clothes also determine the exposure dose. These findings indicate an inadequate knowledge of how to use pesticides properly, unawareness of the potential health impacts, and how to manage the exposure. We recommend the administrative control through comprehensive training on pesticide use and mentoring for farmers. In addition, we also encourage the use of proper PPE, particularly dermal protector, and proper work clothes during pesticide handling to reduce the exposure level.

## Data Availability

The datasets used and/or analysed during the current study are available from the corresponding author on reasonable request.

## Abbreviations

CEL: Cumulative Exposure Level
CPF: Chlorpyrifos
EBDC: Ethylene-bis-dithio-carbamate
IL: Intensity Level of pesticide exposure
OP: Organophosphate
PPE: Personal Protective Equipment
